# Bad Healthy State Compress Temporal Extension Both in Past and Future Orientations

**DOI:** 10.3389/fpsyg.2018.01703

**Published:** 2018-09-11

**Authors:** Jia Zhou, Xingping Han, Juan Fan, Pan Feng, Jingjing Song, Guangyu Jiang, Yong Zheng

**Affiliations:** ^1^Center for Studies of Education and Psychology of Ethnic Minorities in Southwest China, Southwest University, Chongqing, China; ^2^School of Humanities and Management Science, Southwest Medical University, Luzhou, China; ^3^Department of Oncology, Affiliated Hospital of Southwest Medical University, Luzhou, China; ^4^Faculty of Psychology, Southwest University, Chongqing, China

**Keywords:** cancer, health state, temporal extension, long duration, magnitude estimation method

## Abstract

We examined how different health states and cancer-related depression influence time perception in temporal extension (TE) regarding the past and the future over individuals’ entire lives. We used the magnitude estimation method to investigate TE (past and future) and long duration (20 years), and directly measured patients with cancer and their subjective feelings about their lives. In Experiment 1, we investigated whether there were differences in perceptions of TE between patients with cancer (*n* = 144) compared to a healthy control group (*n* = 208). Results indicated that the patients with cancer group evaluated longer TE in healthy states (imagination condition) than in unhealthy states (objectivity condition), and the healthy control group evaluated longer TE in healthy states (objectivity condition) than in unhealthy states (imagination condition). Moreover, the patients with cancer group evaluated longer past-oriented TE than future-oriented TE, whereas the healthy control group evaluated longer future-oriented TE than past-oriented TE. Experiment 2 was conducted to further examine the unexplained findings of Experiment 1. Results indicated that because of the impact of depression on the cancer group, their embodied time system slowed down, and explicit behavior indicated an over-evaluation of time, resulting in group differences. In conclusion, TE could be affected by different health states. Moreover, healthy and unhealthy states may be more associated with future and past orientation, respectively. Lastly, individuals’ time perception can be influenced by depression.

## Introduction

Cancer is a life-threatening disease, and the association between cancer and death is strong (Cox et al., 2007). For many patients, a cancer diagnosis implies a sudden and direct confrontation with the possibility of death that compels them to cope with loss of control of their lives, depression, hopelessness, and other negative experiences. When faced with the distress of cancer, patients’ embodied time system changes. The embodied time system is a crucial inner part of time perception, involving a personal, subjective, and internal process (Lovgren and Hamberg, 2010). Time perception can be defined as the orientation of the individual toward the past, present, and future, in view of a continuously changing present (Sannen, 1998).

Time perception can reflect and be used to assess the distress of a cancer patient (van Laarhoven et al., 2011). Patients with cancer cannot help asking themselves consciously or unconsciously how much time they have left. The immediate reaction of patients with cancer is that their life expectancy has decreased, and the more shorter their lives feel, the more distressed they become (Bölter et al., 2010). Terminally ill patients with cancer reported a shorter future temporal perspective compared to a healthy control group (Fitzpatrick, 1980). One phenomenon—future time perspective (FTP)—describes the relative perception of the length of the future compared to one’s lifetime. FTP is a future-oriented temporal extension (TE). TE that describes relative length of the perception of the past, present, and future compared to one’s lifetime is an integral aspect of time perception (van Laarhoven et al., 2011), and is a linear conception of time including future-oriented, past-oriented, and present-oriented perceptions.

The concept of FTP that stems from Lewin (1939) is an integral part of socio-emotional selectivity theory (SST) (English and Carstensen, 2016), which develops and deepens the domain of TE. SST indicates that poor health results in a more limited FTP predicts that people will experience more negative emotions in the future (Mcwhinney, 1970; Blewett, 1992; Pinquart and Silbereisen, 2006; Sullivan-Singh et al., 2015). Further, Terror Management Theory (TMT) also indicates that the conscious experience of death triggers the potential for anxiety, fear, and depression (Greenberg et al., 1986). Greenberg, Pyszczynski, and Solomon also proposed the dual-process theory of proximal and distal defense, which is an extension of TMT. Dual-process theory specifies distinct defensive systems that deal with conscious and unconscious aspects of death (Pyszczynski et al., 1999). SST is an extension of the proximal defense, which indicates that mortality salience can lead to changes in FTP (English and Carstensen, 2016).

Socio-emotional selectivity theory typically focusesd on future-oriented TE changes, and only a few studies have addressed the past-oriented TE. In a seminal study, Bayes and colleagues tested the degree of suffering among patients with cancer using the perception of past time and found that subjective length of past time correlated strongly with subjective suffering (Bayés et al., 1997). Bayes and colleagues asked patients to assess their current condition by answering the question, “How long did yesterday seem to you?” The responses were scored using a 5-point Likert, ranging from 1 (*very short*) to 5 (*very long*). When the day was perceived as long or very long, their current condition was considered *passable*, *bad*, or *very bad*; however, when the day was perceived as short or very short, their current condition was rated *good* or *very good*. They used a relatively short experience duration (e.g., 1 day) to reflect a specific current distress state; however, Bayes and colleagues did not explore perspective changes among patients with cancer concerning their entire past.

Evidence suggests that cancer is a death-related event and the disease changes people’s future-oriented TE and short-term past estimation change (Lewin, 1939; Bayés et al., 1997; Carstensen and Fredrickson, 1998; English and Carstensen, 2016); moreover, cancer produces diverse kinds of psychological distress that further change individuals’ time perception (Katon and Sullivan, 1990; Grassi et al., 1997; Yang et al., 2013). Mcwhinney defined psychopathological changes in time perception as the “slowing down or inhibition of lived time in depression” (Mcwhinney, 1970; Ghaemi, 2007). Therefore, in the present study, we also consider one of the most commonly encountered types of distress—depression—in patients with cancer. People experiencing depression perceive time as passing more slowly (Thönes and Oberfeld, 2015), accompanied by hopelessness regarding the future, while simultaneously clinging to the past (Ghaemi, 2007; Mendlewicz, 2010). However, cancer-related depression differs from general depression (Pokorney and Bates, 2017). People acquire cancer-related depression after being diagnosed with cancer (Ritterband and Spielberger, 2001). Therefore, it is assumed that if cancer can be completely cured and will not recur, the cancer-related depression will disappear and the embodied time system will perhaps return to its original state. In general, although different emotions and health states can independently influence a person’s embodied time system, how different health states and cancer-related depression influence time perception of the TE regarding the past and future over one’s entire life and not only the future and short-term past remains unclear.

In this study, we were interested in examining the length of time that participants projected themselves back into the past to re-experience their whole lives mentally and into the future to imagine their remaining time. To investigate whether this subjective TE experience was influenced by different health and depression states, we developed a new experimental paradigm that modified the question in Bayes and colleagues’ work to “How long does your past/future seem to you?” We also used the magnitude estimation method (Stevens and Marks, 1980) to record patients’ reflections. Typically, previous researchers used diverse questionnaires to measure TE, and few studies used the magnitude estimation method to directly evaluate or project one’s entire life (Carstensen et al., 1999; Kottergrühn and Smith, 2011).

We selected patients with cancer to represent individuals with a negative health status, who were then compared to a healthy control group. Although an individual’s life consists of the past, present, and future, we did not address the present because it is treated as a time point. Our experiment comprised two parts. Experiment 1 comprised TE assessment of future-oriented and past-oriented extension; Experiment 2 comprised long-duration (LD; 20 years) assessment of future-oriented and past-oriented extension. Experiment 2 comprised supplementary research on the problems that could not be explained in Experiment 1.

Based on existing literature, we proposed five hypotheses: (1) All participants will evaluate longer future-oriented and past-oriented TE in the healthy states than in the cancer disease state; (2) The patients with cancer group will evaluate longer past-oriented TE than future-oriented TE, whereas the healthy control group will evaluate longer future-oriented TE than past-oriented TE; (3) All participants will evaluate longer future-oriented and past-oriented LD in the healthy states than in the cancer disease state; (4) The patients with cancer group will evaluate longer past-oriented LD than future-oriented LD, whereas the healthy control group will evaluate longer future-oriented LD than past-oriented LD; and (5) The more depression experienced, the longer the TE and LD would be.

## Experiment 1

### Method

#### Participants

The study sample included two groups: a patients with cancer group and a healthy control group. Patients with cancer were recruited from the Department of Oncology at Southwest Medical University. Eligible participants were those who had been clinically diagnosed with any cancer and were aware of their disease status. The exclusion criteria were as follows: (1) experienced recurrence or metastasis, (2) treated for cancer for less than a year, and (3) aged < 18 years. The control group participants were recruited from the community through activities publicizing community health knowledge. The exclusion criteria for the control group were as follows: (1) unhealthy, with cancer or other serious diseases; and (2) aged < 18 years. All participants were then screened using a subjective health-related question (“In general, how good have you felt about yourself in the past month?”). Participants responded on a 7-point scale ranging from *very poor* (1) to *very excellent* (7). Patients with cancer who rated their health as 1 or 2, and healthy individuals who rated their health as 6 or 7 were included in the study. Finally, we selected 352 eligible adult participants (age range = 20–82 years, *M* = 45.49, *SD* = 14.00, 71.6% women). The experimental group consisted of 144 patients with cancer (age range = 20–82 years, *M* = 50.56, *SD* = 12.07, 62.5% women). The control group included 208 healthy community-dwelling adults (age range = 20–80 years, *M* = 41.99, *SD* = 14.19, 77.9% women). Participants in the patient and control group did not differ significantly in age, educational background, or socio-economic status.

#### Ethical Considerations

This study was approved by the Institutional Review Board of Southwest University and the Affiliated Hospital of Southwest Medical University and the reference number is XNYD2017268. All participants received an explanation of the study purpose, procedures, benefits, rights to confidentiality, and rights to withdraw. Additionally, all participants provided written, informed consent in adherence with the human participants’ guidelines of the Institutional Ethics Committee. After completing the experiments, participants were provided with a gift (a set of bowls valued at 20 RMB) and a monetary reward (200 RMB) to express our gratitude for their participation.

#### Materials

##### Questionnaire

The subjective health-related question (see above) was administered during initial screening and during the test session to confirm two things: first, patients with cancer could further clarify the subjective evaluation of their own health status; and, second, it ensured that healthy adult participants provided a positive subjective assessment of their health. This question was introduced in detail in 2.1.1. Participants.

Two cancer-related depression questions were used to confirm that cancer was the key reason for the current depression. Patients with cancer were asked the following question: “If your cancer is completely cured and will not recur, will all of your depression go away?” Patients with cancer responded “*yes*” or “*no*.” Healthy participants were asked the following question: “If you get cancer right now, will you experience long-term depression?” Healthy participants responded “*yes*” or “*no*.”

##### Experimental computer

The TE assessment task was programmed and presented by E-prime (Psychology Software Tools Inc., Pittsburgh, PA, United States, vs. 1.0) on a portable ASUS computer with a 60 Hz, 17.3-inch color monitor. E-prime software provides a sensitive measure for reaction times.

##### TE assessment task (**Figure 1**)

**FIGURE 1:**
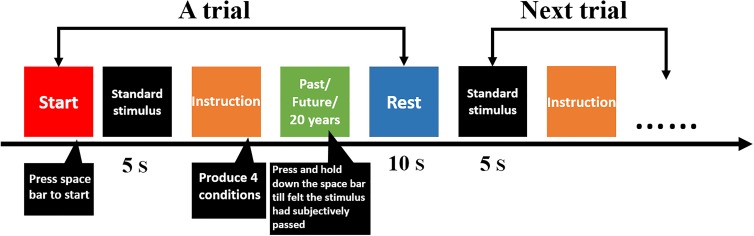
Flow diagram of temporal extension and the long-duration assessment task.

At the beginning of the experiments, researchers presented a standard stimulus—a sound that lasted 5 s—without telling participants that the length of this sound was 5 s. Experimenters indicated that participants felt and remembered the duration that the sound lasted for, and they told participants that the length duration of the sound represented 5 years.

The following test instructions were displayed on the computer screen. Based on the instructions, participants were divided into different health state conditions: objective health status (OHS) and imaginative health status (IHS). For OHS, both the patients with cancer and healthy group were provided the same two instructions: (a) “According to your current health situation, please take the standard stimulus as the baseline, and mentally re-experience your past. The past comprises your birth until now;” and (b) “According to your current health situation, please take the standard stimulus as the baseline, and imagine your future. The future comprises now until your death.” These instructions generated the cancer objectivity condition and the healthy objectivity condition.

For the IHS, instructions also included two sentences that differed slightly for the patients with cancer and healthy group. For patients with cancer, the following sentences were included: (a) “Imagining that you are cured of cancer, please take the standard stimulus as the baseline and mentally re-experience your past. The past comprises your birth until now;” and (b) “Imagining that you are cured of cancer, please take the standard stimulus as the baseline and imagine your future. The future comprises now until your death.” For healthy participants, the following sentences were included: (a) “Imagining that you have cancer, please take the standard stimulus as the baseline and mentally re-experience your past. The past comprises your birth until now;” and (b) “Imagining that you have cancer, please take the standard stimulus as the baseline and imagine your future. The future comprises now until your death.” These instructions produced the healthy imagination condition and the cancer imagination condition. The four conditions were classified into two healthy states—the healthy imagination condition and the healthy objective condition—and two cancer disease states—the cancer imagination condition and the cancer objective condition.

For the same cancer patient, he or she need to complete four trails corresponding to four conditions [(1) objective health status—re-experience your past, (2) objective health status—imagine your future, three imaginative health status—re-experience your past, and (4) imaginative health status—imagine your future). Under each condition, there was only one trail. For the same healthy participant, he or she also need to complete four trails corresponding to four conditions.

All participants sat upright in front of the computer monitor (55 cm distance to screen). The space bar was used as the response button. Participants pressed the space bar by using the index finger of their dominant hand after instructions were displayed on the screen and continuously held down the space bar till they felt subjectively that the past and future had passed. The computer recorded the duration that the spacebar was pressed. The assessment values were recorded by time; therefore, the unit of the dependent variable was seconds. Participants took a short break in the middle of the experiment.

### Preliminary Data Analysis

#### Group Characteristics

Examination of the subjective health-related question scores asked at the beginning of the experiment confirmed that the healthy control group (*M* = 6.51, *SD* = 0.50) had significantly higher subjective health scores than did the patients with cancer group (*M* = 1.33, *SD* = 0.47), *t*[76] = 99.04, *p* < 0.001.

#### Cancer-Related Depression Questions

Regarding cancer-related depression questions, all patients with cancer chose “*yes*,” and all healthy participants chose “*yes*.” This result was the foundation for the design of the IHS conditions.

For data analyses, age was treated as a constant because it could affect the study results. As we aimed to research the influence of health status and health impairment status on TE evaluation, analyses of covariances were performed in both experiments for age. The results for the covariate effects of age were as follows: (*F*(1,349) = 0.013, *p* = 0.908, η^2^ = 0.000) in Experiment 1, and (*F*(1,349) = 1.067, *p* = 0.302, η^2^ = 0.003) in Experiment 2.

### Results

Experiment 1 utilized a 2 × 2 × 2 experimental design. The following were the independent variables: OHS (cancer objectivity condition vs. healthy objectivity condition), IHS (healthy imagination condition vs. cancer imagination condition), and TE (future-oriented vs. past-oriented). The dependent variables were participants’ subjective assessments of the past and the future in the four conditions mentioned above. The greater the value, the longer the time felt in seconds.

**FIGURE 2:**
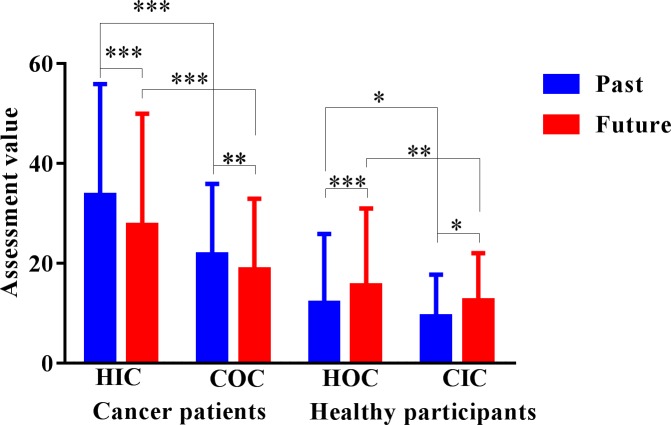
Temporal extension assessment task results. HIC, healthy imagination condition; COC, cancer objectivity condition; HOC, healthy objectivity condition; CIC, cancer imagination condition. The unit of the dependent variable is seconds. ^∗^*p* < 0.05, ^∗∗^*p* < 0.01, ^∗∗∗^*p* < 0.001.

We examined OHS, IHS, and TE by conducting a repeated-measures analysis of variance (ANOVA). The main effects of the three independent variables and interaction were as follows: OHS (*F*(1,349) = 96.90, *p* < 0.001, η^2^ = 0.217); IHS (*F*(1,349) = 6.23, *p* < 0.05, η^2^ = 0.018); TE (*F*(1,349) = 11.46, *p* < 0.01, η^2^ = 0.032); and OHS × IHS × TE, (*F*(1,349) = 4.16, *p* < 0.05, η^2^ = 0.012). Subsequently, we examined the simple effects of the three independent variables using Bonferroni correction, which are reported in the subsequent sections. The means and standard deviations for all the results are presented in **Table 1** and **Figure 2**.

**Table 1:** Means and standard deviations of the temporal extension assessment task and the long-duration assessment task.

Participants	Condition	*M*/*SD*	Past	Future	Past 20 years	Next 20 years
Patients with cancer (*n* = 144)	Healthy imagination condition	*M*	33.68	27.64	23.57	19.59
		*SD*	22.26	22.33	16.82	12.58
	Cancer objectivity condition	*M*	21.71	18.71	23.38	18.68
		*SD*	14.19	14.22	16.42	12.88
Healthy participants (*n* = 208)	Healthy objectivity condition	*M*	12.02	15.55	15.25	16.71
		*SD*	13.86	15.41	6.73	6.58
	Cancer imagination condition	*M*	9.33	12.60	15.13	15.90
		*SD*	8.40	9.42	4.03	4.10

*Healthy imagination condition means that patients with cancer imagine getting healthy and in the imaginative condition to assess the tasks, cancer objectivity condition means that patients with cancer assess the tasks in their current health situation, healthy objectivity condition means that healthy participants assess the tasks in their current health situation, and cancer imagination condition means that healthy participants imagine getting cancer and in the imaginative condition to assess the tasks. The unit of the dependent variable is seconds.*

#### Hypothesis 1

The results indicated that patients with cancer evaluated future-oriented TE as significantly longer in the healthy imagination condition (*M* = 27.64, *SD* = 22.33) than in the cancer objectivity condition (*M* = 18.71, *SD* = 14.22), *p* < 0.001, 95% *CI* = [6.08, 11.40], and past-oriented TE as significantly longer in the healthy imagination condition (*M* = 33.68, *SD* = 22.26) than in the cancer objectivity condition (*M* = 21.71, *SD* = 14.19), *p* < 0.001, 95% *CI* = [9.38, 14.94]. Healthy participants evaluated future-oriented TE as significantly longer in the healthy objectivity condition (*M* = 15.55, *SD* = 15.41) than in the cancer imagination condition (*M* = 12.60, *SD* = 9.42), *p* < 0.01, 95% *CI* = [0.89, 5.29], and past-oriented TE evaluation as significantly longer in the healthy objectivity condition (*M* = 12.02, *SD* = 13.86) than in the cancer imagination condition (*M* = 9.32, *SD* = 8.40), *p* < 0.05, 95% *CI* = [0.27, 4.86]. The within-participant results revealed that, in the two healthy states, participants evaluated longer future-oriented and past-oriented TE than in the two unhealthy states.

However, there were contrary results in the OHS condition in the between-participants analyses. Future and past-oriented TE were evaluated to be significantly longer by the patients with cancer group (*M*_future_ = 18.71, *SD*_future_ = 14.22; *M*_past_ = 21.71, *SD*_past_ = 14.19) than the healthy control group (*M*_future_ = 15.55, *SD*_future_ = 15.41; *M*_past_ = 12.02, *SD*_past_ = 13.86; *p* < 0.001). The between-participant results obtained in the OHS condition indicated that the patients with cancer group evaluated future and past-oriented TE to be longer in the unhealthy states compared to the healthy control group in the healthy states.

In the IHS condition, this part of the between-participant results was consistent with the within-participant results. The patients with cancer group evaluated future and past-oriented TE evaluation to be significantly longer (*M*_future_ = 27.64, *SD*_future_ = 22.33; *M*_past_ = 33.68, *SD*_past_ = 22.26) than did the healthy control group (*M*_future_ = 12.60, *SD*_future_ = 9.42; *M*_past_ = 9.33, *SD*_past_ = 8.40; *p* < 0.001).

#### Hypothesis 2

Under both the OHS and IHS, the patients with cancer group evaluated past-oriented TE (*M*_OHS_ = 21.71, *SD*_OHS_ = 14.19; *M*_IHS_ = 33.68 *SD*_IHS_ = 22.26) to be significantly longer than future-oriented TE (*M*_OHS_ = 18.71, *SD*_OHS_ = 14.22; *M*_IHS_ = 27.64, *SD*_IHS_ = 22.33; *P*_OHS_ < 0.01, 95% *CI*_OHS_ = [0.70,3.31]; *P*_IHS_ < 0.001, 95% *CI*_IHS_ = [2.51,8.35]). Contrarily, in the healthy control group, future-oriented TE was evaluated to be significantly longer (*M*_OHS_ = 15.55, *SD*_OHS_ = 15.41; *M*_IHS_ = 12.60, *SD*_IHS_ = 9.42) than past-oriented TE (*M*_OHS_ = 12.02, *SD*_OHS_ = 13.86; *M*_IHS_ = 9.33, *SD*_IHS_ = 8.40; *P*_OHS_ < 0.05, 95% *CI*_OHS_ = [0.70,5.52]; *P*_IHS_ < 0.001, 95% *CI*_IHS_ = [1.51,3.6]). These results revealed that the patients with cancer group considered past-oriented TE to be longer than future-oriented TE, whereas the healthy control group evaluated future-oriented TE to be longer than past-oriented TE.

### Discussion

In Experiment 1, our findings imply that different health states can change the length of individuals’ subjective TE. In patients with cancer, when the patients imagined that their illness was completely cured, their future TE became longer than in the OHS condition. It was easy to understand why they thought that curing the disease would increase their life expectancy. When healthy participants imagined themselves getting cancer, their future TE became shorter than in the OHS condition. It is also easy to understand why participants thought that future TE would be shortened by a life-threatening disease (Vrinten et al., 2016). TMT indicated that mortality salience has led to strong limited FTP. Contrastingly, if the thought of death was removed from consciousness, individuals’ future time will be longer (Greenberg et al., 1986; Pyszczynski et al., 1999).

In addition to these predicted findings, more unexpected effects were obtained for past-oriented TE; in the IHS condition, patients with cancer exhibited longer past TE, and the control group exhibited a shorter past TE, respectively (compared to the OHS condition). Notably, life-threatening cancer not only changed individuals’ subjective perceptions about future-oriented TE, it also made past-oriented TE shorter. We initially believed that past-oriented TE was an already experienced duration, that it differed from imagined future TE, and that it would remain constant in participants’ minds. However, unpredictably, it changed with changes to health status (OHS and IHS). This implies that a poor health state made TE shorter, whereas a good health state made it longer. This result can explain the common phenomenon of why a 40-year-old patient with cancer finds the 40 years that have passed to be too short or why a 70-year-old patient with cancer feels that their past life was shorter than they would have wanted it to be. Of note, all the above of the results are within-group comparisons.

Nevertheless, contrary results were obtained in between-participant analyses. In the OHS condition, patients with cancer evaluated TE as significantly longer in both orientations compared with the healthy control group. There are two possible reasons for this. Previous studies reported that perceived slowing down of time is very characteristic of depression (Massie and Holland, 1990; Mendlewicz, 2010; Ratcliffe, 2012; Thönes and Oberfeld, 2015) and that depressive symptoms are often an appropriate and normal reaction to a life-threatening event including having cancer (Schroevers et al., 2003). Another reason was that patients with cancer were eager to prolong life. Consequently, it is possible that the evaluation of TE in patients with cancer stemmed from their desire to live; thus, they evaluated TE to be longer than did healthy participants. It is not only health state alone that influences time evaluation; therefore, Experiment 2 was conducted to further examine the unexplained findings of Experiment 1.

Additionally, we investigated the two orientations of TE (OHS and IHS conditions) in patients with cancer and healthy participants. Results indicated that patients with cancer indicated a significantly shorter future TE than past TE; in contrast, healthy participants indicated a significantly longer future TE than past TE. One explanation was that people with cancer feel uncertain about their future, which makes them experience a sense of despair and loss of hope (Everson et al., 1996; Schulz et al., 1996; Esmaeili et al., 2015), while healthy participants who do not have a life-threatening disease have a bright and hopeful future. Even in the IHS condition, participants’ perception about their future cannot be easily changed. SST indicates that most older adults and people with a serious illness are more present-focused and less future-focused than are younger and healthy people (Mcgee and Barker, 1982; Shmotkin, 1991). Routledge et al. (2008), who examined nostalgia, argued that remembering the past can provide meaning to one’s life and help manage existential concerns. Nostalgia was an effective defense mechanism to eliminate or reduce death thoughts (Routledge et al., 2008).

## Experiment 2

This study was designed to further examine the unexplained findings of Experiment 1. In Experiment 2, the first objective was to control people’s desire to live longer to not interfere with the results of the experiment. The second objective was to examine whether depression can affect individuals’ LD perception.

### Method

#### Participants

All participants were the same as in Experiment 1.

#### Materials

##### Questionnaire

The Beck Depression Inventory (BDI) was used to measure the level of depressive symptoms in patients with cancer and healthy participants. The BDI is one of the most widely used psychometric methods for detecting depression in normal populations and in diverse psychiatric patient cohorts (Richter et al., 1998). All participants completed the 21-item BDI, which contains four statements each and describes the intensity of a particular depressive symptom (Beck et al., 1967; Beck and Beck, 1973).

##### Experimental computer

The same computers were used as in Experiment 1.

##### LD assessment task

The procedure was identical to the TE assessment task in Experiment 1, except that “past time” and “future time” in the instructions were revised to “the past 20 years” and “the next 20 years.” We chose 20 years for three reasons: one was to ensure that people’s desire for life did not interfere with the experimental results; the second was that 20 years was a long duration; and the third was that the youngest participants were 20-years-old (see **Figure 1**).

### Results

In Experiment 2, we utilized a 2 × 2 × 2 experimental design. The following were the independent variables: OHS × IHS × LD (future 20 years vs. past 20 years). The dependent variables were participants’ subjective assessments of the past and next 20 years in the four conditions mentioned above.

**FIGURE 3:**
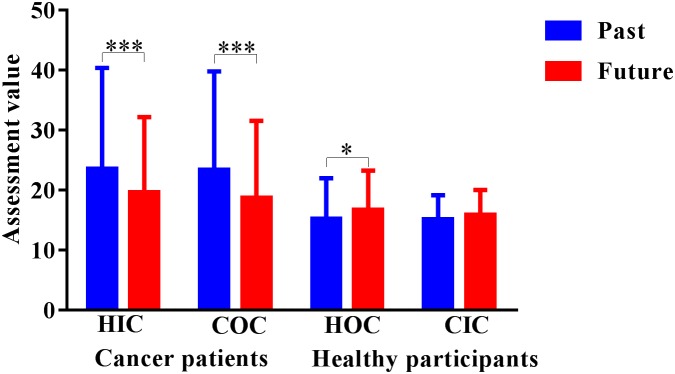
Long-duration assessment task results. HIC, healthy imagination condition; COC, cancer objectivity condition; HOC, healthy objectivity condition; CIC, cancer imagination condition. The unit of the dependent variable is seconds. ^∗^*p* < 0.05, ^∗∗^*p* < 0.01, ^∗∗∗^*p* < 0.001.

We examined OHS, IHS, and LD using a repeated measures ANOVA. The main effects of the three independent variables and interaction were as follows: OHS (*F*(1,349) = 42.72, *p* < 0.001, η^2^ = 0.109); IHS (*F*(1,349) = 0.029, *p* = 0.864, η^2^ = 0.000); TE (*F*(1,349) = 7.27, *p* < 0.01, η^2^ = 0.02); and OHS × IHS × TE, (*F*(1,349) = 0.055, *p* = 0.814, η^2^ = 0.000). The main effect of IHS was not significant, and there was no interaction between the three independent variables (OHS × IHS × LD). Only one two-way interaction was significant: OHS × LD (*F*(1,349) = 41.87, *p* < 0.001, η^2^ = 0.107). The means and standard deviations for all results are presented in **Table 1** and **Figure 3**.

#### Hypothesis 3

The results indicated that in the patients with cancer group, there were no significant effects of the past 20 years evaluation in the healthy imagination (*M* = 23.57, *SD* = 16.82) or cancer objectivity conditions (*M* = 23.38, *SD* = 16.42), and there was also no significant effect of the next 20 years evaluation in the healthy imagination (*M* = 19.59, *SD* = 12.58) or cancer objectivity conditions (*M* = 18.68, *SD* = 12.88). The healthy control group exhibited the same results; there were no significant effects of the past 20 years evaluation in the healthy objectivity (*M* = 15.25, *SD* = 6.73) or cancer imagination conditions (*M* = 15.13, *SD* = 4.03), and there was also no significant effect of the next 20 years evaluation in the healthy objectivity (*M* = 16.71, *SD* = 6.58) or healthy objectivity conditions (*M* = 15.90, *SD* = 4.10) (**Figure 3**).

#### Hypothesis 4

The results revealed that in both the OHS and IHS conditions, in the patients with cancer group, there was significantly longer past 20 years evaluation (*M*_OHS_ = 23.38, *SD*_OHS_ = 16.42; *M*_IHS_ = 23.57, *SD*_IHS_ = 16.82) than future-20 years evaluation (*M*_OHS_ = 18.68, *SD*_OHS_ = 12.88; *M*_IHS_ = 19.59, *SD*_IHS_ = 12.58); *P*_OHS_ < 0.001, 95% *CI*_OHS_ = [2.69,6.96]; *P*_IHS_ < 0.001; 95% *CI*_IHS_ = [2.35,6.50]. Contrastingly, for the OHS condition in the healthy control group, there was significantly longer future-20 years evaluation (*M*_OHS_ = 16.71, *SD*_OHS_ = 6.58) than the past 20 years evaluation ((*M*_OHS_ = 15.25, *SD*_OHS_ = 6.73); *P*_OHS_ < 0.05, 95% *CI*_OHS_ = [0.05,3.48]). The results indicated that the patients with cancer group evaluated the past 20 years to be longer than the next 20 years, whereas the healthy control group evaluated the next 20 years to be longer than the past 20 years in the OHS condition. There was no significant difference between the next 20 years (*M*_IHS_ = 15.90, *SD*_IHS_ = 4.10) and the past 20 years (*M*_IHS_ = 15.13, *SD*_IHS_ = 4.03) in the IHS condition in the healthy control group.

#### Hypothesis 5

The main effect of IHS was not significant; however, OHS × LD interactions were significant: (*F*(1,349) = 41.87, *p* < 0.001, η^2^ = 0.107). We examined the simple effects of the two variables using Bonferroni correction, and the means and standard deviations were not presented in **Table 1**. There was a significantly longer LD evaluation in the patients with cancer group (*M*_past_ = 23.48, *SD*_past_ = 12.83; *M*_future_ = 19.13, *SD*_future_ = 9.49) than in the control group (*M*_past_ = 15.19, *SD*_past_ = 5.12; *M*_future_ = 16.30, *SD*_future_ = 5.08); *P*_past_ < 0.001, 95% *CI*_OHS_ = [6.23,10.31]; *P*_future_ < 0.01, 95% *CI*_OHS_ = [0.73,3.95]. To examine the differences in depression, two independent sample *t*-tests were performed for the patients with cancer group and the healthy control group. The results revealed significantly higher depression ratings in the patients with cancer group (*M* = 13.88, *SD* = 11.17) than in the healthy control group (*M* = 9.39, *SD* = 8.12), *t*[247] = 4.49, *p* < .001. To examine the correlation between depression and LD, we performed Pearson’s correlations for both groups. The results showed that the change in the past 20 years LD evaluation (*r* = 0.46, *p* < .001) and future-20 years LD evaluation (*r* = 0.39, *p* < .001) were positively correlated with the total depression score in the patients with cancer group. In the healthy control group, there were no significant correlations. The results revealed that the higher the degree of depression, the longer the duration assessed by the participants.

### Discussion

The findings of Experiment 2 revealed that different health states cannot change the length of LD within-group comparison. The assignment of LD was relatively constant for each participant in different health conditions, perhaps because 20 years was a specific period, whereas TE (life expectancy) was an unspecified duration. The design of Experiment 2 achieved its purpose of controlling people’s desire to live longer.

We investigated the two orientations of LD in OHS and IHS conditions in patients with cancer and healthy participants. In line with Experiment 1, our results also indicated that patients with cancer exhibited a significantly shorter future LD than past LD, and, contrarily, healthy participants exhibited a significantly longer future LD than past LD in the OHS condition. However, if the control group imagined getting cancer, there was no significant difference between the past and the next 20 years. Patients with cancer indicated lack of confidence in the future and were immersed in past memories, whereas the control group was confident in the future in the OHS condition, and looked toward it (Zimbardo and Maslach, 1977; Brannen and Nilsen, 2002). However, in the IHS condition, participants’ confidence in the future had decreased.

In between-participant comparisons, patients with cancer evaluated significantly longer LD than did the control group. This result was the same as that of Experiment 1. The cancer group had a significantly higher depression score than did the control group, and the higher the cancer depression score, the longer the LD evaluation (significantly positively correlated). In line with previous studies, the results suggest that depression was a key factor that affected participants’ time perception. Depression destroyed people’s embodied time systems (Thönes and Oberfeld, 2015), and their systems indicated slower internal time experiences (Bech, 1975; Blewett, 1992; Mioni et al., 2016). Both Experiments 1 and 2 revealed that the evaluation times of TE and LD in the cancer group were longer than in the control group, and it was further revealed that depression was a relatively stable variable in influencing participants’ time assessment. Because of the impact of depression on the cancer group, the embodied time system slowed down, and explicit behavior indicated an over-evaluation of time, leading to system differences between the groups.

## General Discussion

Two experiments were performed to examine the relationships among OHS, IHS, TE, LD, and depression in patients with cancer and healthy participants. The magnitude estimation method allowed us to investigate TE and LD; moreover, it enabled people to directly project their subjective feelings of long periods rather than indirect responses to questionnaires. Therefore, we tested the idea that different health states and depression may function as explanatory variables for observed time perception differences. The main conclusions from these experiments were as follows: (1) TE could be affected by different health states; (2) healthy states are associated more with future orientation and unhealthy states are associated more with past orientation (except the control group in the IHS condition in Experiment 2); and (3) individuals’ time perception can be affected by depression. These conclusions are discussed below.

First, previous studies concerning the nature of time perception indicated that people with life-threatening diseases were characterized by a limited future perspective (Carstensen and Fredrickson, 1998; Pinquart and Silbereisen, 2006); however, they did not mention the impact on the past. In the present study, we investigated both future and past orientations of TE in different health states. For unhealthy (patients with cancer in OHS, healthy control group in IHS) within-group comparison, the estimate of the future became shorter among the participants, and, more interestingly, the past became shorter as well. However, in the healthy conditions (patients with cancer in IHS, healthy control group in OHS), the estimate of the future and past became longer. Therefore, individuals’ TE was affected by different health conditions and dynamic changes. Patients with cancer were in a compressed TE state, experiencing a compressed future and past. In other words, young people who have cancer feel that their life expectancy has been shortened drastically, with the feeling becoming stronger the younger they are. Elderly people with cancer also feel that their life expectancy has been shortened subjectively, but not as strongly as young people do. Accordingly, under the influence of such TE perception, younger patients with cancer reported more psychological distress compared with older patients with cancer (Bates, 2011; Krok et al., 2013). However, whether healthy or seriously ill, people’s perceptions of the specific 20-year duration were relatively constant, sensible, and calm, although 20 years was long duration. This indicates that LD evaluation was unrelated to health states.

Second, the patients with cancer group and healthy control group differed in their subjective perceptions of the past and future (including TE and LD). The past time assessment among patients with cancer was not only significantly longer than future time in the OHS condition, it was longer in the IHS condition. This occurred because individuals were nevertheless required to face an objective reality where they experienced the objective consequences of cancer. They not only lacked confidence about the future (Schulz et al., 1996), they were afraid of the future (Crist and Grunfeld, 2013). Even if the disease is under control, most patients with cancer express the fear of cancer recurrence (Cella et al., 1990; Crist and Grunfeld, 2013). Due to uncertainty and fear about the future, patients with cancer tend to be more immersed in memories of the past and present (Dousti et al., 2013). Moreover, it is the fear of and lack of confidence in the future that probably causes patients with cancer to feel uneasy when imagining their future. TMT provided a new perspective that nostalgia was an effective defense mechanism to eliminate or reduce death thoughts (Routledge et al., 2008), which could account for the finding that the assessment of the past is longer than that of the future among patients with cancer. In contrast, the healthy control group feels they have a bright and hopeful future, which probably causes them to imagine a more detailed future.

Finally, one noteworthy question is how the longer assessment of TE and LD among patients with cancer than the control group should be interpreted. Patients with cancer, regardless of OHS or IHS conditions, evaluated LD and TE to be longer than did the healthy group. This reveals that, in addition to the health variables, there was another variable that affected participants’ perception of time. Previous studies observed that clinical depression is prevalent among patients with cancer (Sellick and Crooks, 1999), and many patients experience subthreshold or subsyndromal symptoms without meeting the criteria for a depression diagnosis (Grassi et al., 1996). Previous studies have also indicated that depression affects people’s perception of time (Mendlewicz, 2010; Thönes and Oberfeld, 2015). In the present study, there were significant differences in the depression scores between the two groups, and the time assessment of LD in the cancer group was positively related to depression. This suggests that depression can change individuals’ time perception; accordingly, in patients with cancer, cancer is the source of depression, and LD of depression has gradually changed the internal mechanism of time perception. Therefore, as the internal clock slows, when patients with cancer projected the past and future time by means of magnitude estimation method, the outward manifestation indicated overproduction of time than that of the healthy group. Thus, due to system differences of depression, it becomes easy to understand why patients with cancer assessed longer TE and LD than did healthy participants.

## Limitations and Future Studies

This study had its limitations. First, the two groups were not completely matched in their age and sample size. Our age range was 20–82 years; therefore, matching the age of the two groups was not easy. Further age differences prompted us to control for age using covariates. However, the statistical control was not as good as that of the two groups, and there was no difference in age. In addition, patients with cancer were difficult to recruit, resulting in uneven sample sizes for the two groups. Second, the cancer-related depression questions have their limitations. For example, people do not know how long their depression will last. Further, imagining is obviously distinct from real situations. This caused confusion among participants often (e.g., one said, “I will definitely be depressed; but, I’m not sure how long [it will] last. It may be short, and it may be long”). Differences in the attention paid to time may have affected people’s responses to this question. Third, in Experiment 2, we chose 20 years as a stimulus. This was selected because the youngest participants were 20-years-old, thus satisfying those participants’ assessment of his/her past 20 years. However, in doing so, we did not examine the associations with longer periods. This requires different LD settings for different age groups. Therefore, in future studies, the effect of age on the cognition of TE and LD should be further studied. We should also study how TE and LD change at different ages under the influence of disease. Fourth, we did not control for potential response tendencies due to different cancer types. This means that diverse cancer types might have had distinct effects on people, thus instigating different responses to time perception. Although we screened participants with the subjective health-related question to ensure homogeneity, diverse cancer types may have influenced TE. Finally, we failed to measure the duration of patients’ illness. If the time of illness has been measured, maybe further statistics could be conducted. We can further understand the relationship between the time of illness and time perception. Maybe there was a positive relationship between the both.

Future studies should also examine the temporal cognition of patients with cancer without depression but with other mental disorders (such as anxiety and mania). Previous studies have indicated that, contrary to depression, patients with mania experience time faster (Ghaemi, 2007). Future studies should also incorporate the present orientation. Defining the scope of the past, present, and the future clearly will be a difficult problem that requires an extremely good and innovative experimental design. Future studies should also need to measure the duration of patients’ illness. Finally, researchers should focus on cancer survivors as the next step, preferably using longitudinal studies, and further investigate the temporal cognitive changes of TE after objective health improvements.

### Attachment

#### Magnitude Estimation Method

Stevens and Marks (1980) developed a ratio scale according to Weber’s law (Gibbon, 1977) and defined it clearly as a direct measurement of sensation based on a basic individual development skill that utilizes high-level cognitive processes (Marks, 1988). The specific steps of this method are as follows: the experimenter attempts to present a standard stimulus (e.g., weight) and assigns a subjective value to the standard stimulus (e.g., 10). Participants can treat the subjective value as the standard; then, the subjective strength of other stimuli is represented by other data using 10 as the standard. The “other data” can express the subjective feelings of the participants.

Originally, the magnitude estimation method (Stevens and Marks, 1980) was criticized for its inability to control for several confounding variables, as participants’ assignment of numerical values reflected their habits more than their sensations. In response to this criticism, Stevens revised his approach to use diverse sensory channels and a cross-modality ratio matching method in experimental practice to verify the theory. Overall, using magnitude estimation method enables participants to assess distinct periods and quantize intuitively.

## Author Contributions

JZ designed and conducted the experiment protocol, analyzed the data, and drafted this manuscript. XH and JF participated in conducting the experiments. PF, GJ, and JS participated in the development of the encoding principle and reviewed the manuscript. YZ reviewed the manuscript and provided critical comments and revision. All authors approved the final manuscript.

## Conflict of Interest Statement

The authors declare that the research was conducted in the absence of any commercial or financial relationships that could be construed as a potential conflict of interest.
